# Ternary Liquid–Liquid Equilibria for Mixtures of {Ionic Liquid + Thiophene or Benzothiophene + Heptane} at *T* = 308.15 K

**DOI:** 10.1007/s10953-014-0276-y

**Published:** 2014-12-23

**Authors:** Urszula Domańska, Klaudia Walczak

**Affiliations:** Department of Physical Chemistry, Faculty of Chemistry, Warsaw University of Technology, Noakowskiego 3, 00-664 Warsaw, Poland

**Keywords:** Ionic liquids, 1-Pentyl-1-methylpiperidinium bis{(trifluoromethyl)sulfonyl}imide, Tributylethylphosphonium diethylphosphate, Ternary (liquid–liquid) phase equilibrium, Selectivity, Solute distribution ratio, NRTL correlation

## Abstract

In this work, the desulfurization ability of alkyl-piperidinium-based and phosphonium-based ionic liquids (ILs) for (thiophene or benzothiophene + heptane) mixtures are studied. With this aim, ternary liquid–liquid phase equilibrium data (LLE) have been obtained for mixtures of {IL (1) + thiophene, or benzothiophene (2) + heptane (3)} at *T* = 308.15 K and *p* = 101.33 kPa. For this study 1-pentyl-1-methylpiperidinium bis{(trifluoromethyl)sulfonyl}imide, [C_1_C_5_PIP][NTf_2_], and tributylethylphosphonium diethylphosphate, [P_2,4,4,4_][DEP], were used. The suitability of these ILs as solvents for extractive desulfurization has been evaluated in terms of the solute distribution ratio and selectivity. Immiscibility was observed in the binary liquid systems of (thiophene, or benzothiophene + heptane) with both ILs. One of the studied ILs, [C_1_C_5_PIP][NTf_2_], shows high distribution ratios and high selectivities for extraction of sulfur compounds. The data obtained have been correlated with the non-random two liquid NRTL model. The experimental tie-lines and the phase compositions in mole fractions in the ternary systems were calculated with an average root mean square deviation of 0.0052.

**Electronic supplementary material:**

The online version of this article (doi:10.1007/s10953-014-0276-y) contains supplementary material, which is available to authorized users.

## Introduction

In recent years, the deep desulfurization of diesel fuel has become the most studied process with different techniques (extraction, liquid–liquid separation, oxidative desulfurization, adsorption). The emission of sulfur from petrol and diesel oils, which is linked to acid rain, plays a crucial role in pollution problems of large conglomerates. Thus, the USA and European countries have issued regulations regarding sulfur content in fuels [1, 2]. Due to this situation, the European Union approved a new directive stating that the content of total sulfur in European gasoline and diesel fuels from 2010 onwards must be at a maximum concentration level of 10 ppm [2]. Ionic liquids (ILs) have the ability to extract aromatic sulfur-containing compounds at ambient conditions. Additionally, ILs are immiscible with the fuel, are non-volatile and can be regenerated and recycled by solvent washing. Oxidative desulfurization in future years probably will bring better results than simple liquid–liquid separation, however, first the best ILs must be chosen. At present, the hydrodesulfurization (HDS) processes is the established method used in some industrial technologies to remove organic sulfur from fuels. However, to achieve low sulfur targets with current HDS technology, higher temperatures, higher pressures, larger reactor volumes, and more active catalysts are needed [3]. The HDS process does not purify fuels of polycyclic organic sulfides such as thiophene, benzothiophene, methyldibenzothiophenes, 4,6-dibenzothiophenethiols, thioethers, and disulfides. Therefore, new technologies for deep desulfurization have become necessary. Extraction desulfurization, which has begun to be popular, especially with ILs, has the potential for being an alternative and future complementary technology for deep desulfurization [4–10]. In order to solve this problem, extractive liquid–liquid equilibrium (LLE) desulfurization with ILs has been proposed [7, 10–18].

The 1-alkylpiperidinium-based [18], or pyrrolidinium-based ILs with different anions [15], or 1-alkylcyanopyridinium-based ILs [16], have been recently studied in our laboratory in ternary LLE {IL + thiophene, or benzothiophene + heptane) with high selectivities. Attractive extraction parameters were presented as well for 1-ethyl-3-methylimidazolium bis{(trifluoromethyl)sulfonyl}imide, [EMIM][NTf_2_] ([11], and references cited therein), 1-ethyl-3-methylimidazolium acetate, [EMIM][OAc] [12], 1-ethyl-3-methylimidazolium thiocyanate, [EMIM][SCN] [7], and 1,3-dimethylimidazolium methylphosphonate [DMIM][MP] [7].

This work is a continuation of our systematic studies on the physicochemical properties and the extraction abilities of piperidinium-based ILs ([18] and references cited therein). Proposed by us are new interaction parameters for the group contribution method Modified UNIFAC for the piperidnium-based ILs [19], predicted attractive infinite dilution selectivity, and capacity of piperidinium-based ILs (alkane chain, *n* = 3–6) in the thiophene/heptane separation problem at *T* = 328.15 K.

To our best knowledge, the phosphonium-based IL (tributyl-methylphosphonium methylsulfate, [P_1,4,4,4_][CH_3_SO_4_]) was measured in ternary LLE for the separation of thiophene from cyclohexane at *T* = 298.15 K with very low selectivities in a range of 1.5 to 5.4 [20]. Better results were obtained with deep eutectic solvents, DES, containing phosphonium-based ILs with ethylene glycol. DES is composed of methyltripentylphosphonium bromide, [P_1,5,5,5_][Br], and ethylene glycol (as a hydrogen bond donor) showed selectivities of about *S* = 60–100 for ternary LLE at *T* = 318 K for benzene/hexane separation [21]. Poorer results were estimated with DES composed of tetrabutylphosphonium bromide, [P_4,4,4,4_][Br], and ethylene glycol for the separation of toluene/heptane [22]. Usually, the results of separation processes for aliphatic/aromatic hydrocarbons provide good information for the separation of aliphatic/aromatic sulfur compounds. We can expect similar or even better results for the chosen IL. On the other side, there are very good results obtained with methylphosphonate [7] and diethylphosphate [12] anions of ILs in ternary LLE (IL + thiophene + heptane) mixtures.

In this work we report experimental ternary LLE data for one additional piperidinium-based IL, {1-pentyl-1-methylpiperidinium bis{(trifluoromethyl)sylfonyl}imide [C_5_MPIP][NTf_2_], for comparison with measured earlier 1-propyl, or 1-butyl-, or 1-hexyl-1-methylpiperidinium bis{(trifluoromethyl)sylfonyl}imide [18]. Moreover, tributylethylphosphonium diethylphosphate, [P_2,4,4,4_][DEP] was chosen to check the influence of the anion. The [DEP]^−^ anion in [EMIM][DEP] shows interesting results for thiophene extraction from hexane at *T* = 298.15 K [12]. The solvents heptane, thiophene, and benzothiophene used in this work are model compounds for fuel and sulfur organic hydrocarbons, respectively. The ternary systems {IL (1) + thiophene, or benzothiophene (2) + heptane (3)} were investigated at *T* = 308.15 K and *p* = 101.33 kPa. The experimental tie-lines for four ternary mixtures have been correlated with the NRTL equation [23, 24]. The solute distribution ratio and the extractive selectivity were determined from the experimental data, and are compared to the literature data.

## Experimental

The ILs studied, [C_1_C_5_PIP][NTf_2_] and [P_2,4,4,4_][DEP], were purchased from IoLiTec. The names, abbreviations, structures, measured densities and mass fraction of ILs are listed in Table 1. The names, CAS numbers, sources, mass fraction purities, purification method, water content, and measured and literature densities of all chemicals used are shown in Table 1S in the Supplementary Material Information. Most of chemicals used were from Merck or Sigma Aldrich. The samples of ILs were dried for 24 h at 300 K under reduced pressure to remove volatile impurities and trace amounts of water. Thiophene and benzothiophene were stored over freshly activated molecular sieves of type 4 Å (Union Carbide). The densities for all substances were measured at *T* = 298.15 and 101.33 kPa. The method and uncertainties have been described previously [18].

**Table 1:** List of investigated ionic liquids: structure, name, abbreviation of name, molar mass (*M*) and density

Cation	Anion	Name, abbreviation	M/(g·mol^−1^)	Exp. Density *ρ*/g·cm^−3^ (298.15 K; 101.33 kPa)
		1-Pentyl-1-methylpiperidinium bis{(trifluoromethyl) sulfonyl}imide [C_1_C_5_PIP][NTf_2_]	450.46	1.35016
		Tributylethylphosphonium diethylphosphate, [P_2,4,4,4_][DEP]	384.47	1.0089

The water content was analyzed by the Karl-Fischer titration (method TitroLine KF). The sample of IL, or solvent, was dissolved in methanol and titrated in steps of 0.0025 cm^3^. The error in the water content is ±10 × 10^−6^ in mass fraction for the 3 cm^3^ of injected IL. The water content in solvents used was less than 350 × 10^−6^ in mass fraction.

To obtain the experimental LLE tie-lines, mixtures with compositions inside the immiscible region of the systems were introduced into a jacketed glass cell of volume of 100 cm^3^. The solution was mixed with a coated magnetic stirring bar. The vessel was tightly closed to avoid losses by evaporation or pickup of moisture from the atmosphere. The jackets were connected to a thermostatic water bath (LAUDA Alpha) to maintain a constant temperature of *T* = 308.15 K (±0.05). The mixtures were stirred for 6 h to reach thermodynamic equilibrium and after a minimum of 12 h were analyzed. After the phase separation, samples of about (0.1–0.3) × 10^−3 ^cm^3^ were taken from both phases using glass syringes with coupled stainless steel needles. A sample of the phase was placed in an ampoule with a capacity of 2 × 10^−3 ^cm^3^. The ampoule was closed with a septum cap. Next, acetone (1.0 cm^3^) was added to the samples to avoid phase splitting and to maintain a homogeneous mixture. Propan-1-ol was used as internal standard for the GC-analysis. Because of the low vapor pressure, the ILs used in this work cannot be analyzed by GC. Thus, only thiophene or benzothiophene and heptane were analyzed; the mass fraction of the third component, the IL, was determined by subtracting the mole fractions of the two other components from unity.

The compositions were analyzed by gas chromatography (PerkinElmer Clarus 580 GC equipped with auto sampler and FID and TCD detectors). The capillary column of the chromatograph was protected with a pre-column to avoid the non-volatile ionic liquid reaching the column in the case of a leak from the glass wool in the liner. The TotalChrom Workstation software was used to obtain the chromatographic areas for the thiophene, or benzothiophene, heptane and the internal standard propan-1-ol. Samples were injected three times, and the average value was calculated. Details of the operational conditions of the apparatus are reported in Table 2S in the Supplementary Material. The estimated uncertainty in the determination of mole fraction compositions is ± 0.003 for compositions of the hydrocarbon-rich phase and ±0.005 for compositions of the IL-rich phase.

## Results and Discussion

The equilibrium compositions of the experimental tie-line ends in ternary systems of four mixtures {IL (1) + thiophene or benzothiophene (2) + heptane (3)}, at *T* = 308.15 K and *p* = 101.33 kPa are reported in Table 2. Experimental solubilities for [C_1_C_5_PIP][NTf_2_] and [P_2,4,4,4_][DEP] in heptane at *T* = 308.15 K are totally different from each other. In the binary {IL (1) + heptane (3)} system complete liquid miscibility (solubility of heptane in the IL) is up to mole fraction of heptane $x_{3}^{\text{IL}}$ = 0.089 and $x_{3}^{\text{IL}}$ = 0.456 for [C_1_C_5_PIP][NTf_2_] and [P_2,4,4,4_][DEP], respectively. The solubility of heptane is much larger in [P_2,4,4,4_][DEP] than that in [C_1_C_5_PIP][NTf_2_]. The piperidinium-based IL shows much lower solubility of heptane in the IL. In comparison with piperidinium-based IL measured by us earlier, heptane shows higher solubility in [C_1_C_5_PIP][NTf_2_] than in [C_1_C_3_PIP][NTf_2_] ($x_{3}^{\text{IL}}$ = 0.051, at *T* = 308.15 K [18]). This effect is due to an increase in the van der Waals interactions between the hydrocarbon chain of the cation and heptane.

**Table 2:** Compositions of experimental tie lines, solute distribution ratios, *β*, and selectivity, *S*, for ternary systems {[C_1_C_5_PIP][NTf_2_] or [P_2,4,4,4_][DEP] (1) + thiophene or benzothiophene (2) + heptane (3)} at *T* = 308.15 K, *p* = 101.33 kPa

Hydrocarbon-rich phase		IL-rich phase		*β*	*S*
$x_{1}^{\text{I}}$	$x_{2}^{\text{I}}$	$x_{1}^{\text{II}}$	$x_{2}^{\text{II}}$		
[C_1_C_5_PIP][NTf_2_] + thiophene + heptane					
0.000	0.000	0.911	0.000	–	–
0.000	0.057	0.770	0.142	2.49	26.7
0.000	0.111	0.663	0.250	2.25	23.0
0.000	0.191	0.564	0.352	1.84	17.7
0.000	0.230	0.523	0.392	1.70	15.4
0.000	0.353	0.424	0.490	1.39	10.4
0.000	0.447	0.376	0.542	1.21	8.2
0.000	0.530	0.337	0.582	1.10	6.4
0.000	0.696	0.281	0.650	0.93	4.1
0.000	0.800	0.241	0.702	0.88	3.1
0.000	0.891	0.213	0.750	0.84	2.5
0.000	1.000	0.186	0.814	0.81	–
[C_1_C_5_PIP][NTf_2_] + benzothiophene + heptane					
0.000	0.000	0.911	0.000	–	–
0.000	0.025	0.803	0.109	4.36	48.3
0.000	0.052	0.705	0.210	4.04	45.0
0.000	0.092	0.589	0.326	3.54	37.9
0.000	0.161	0.444	0.472	2.93	29.3
0.000	0.243	0.383	0.531	2.19	19.2
0.000	0.346	0.299	0.611	1.77	12.8
0.000	0.460	0.236	0.674	1.47	8.8
0.000	0.553	0.208	0.696	1.26	5.9
0.000	0.766	0.144	0.758	0.99	2.4
0.000	0.853	0.096	0.819	0.96	1.7
0.000	0.915	0.060	0.873	0.95	1.2
[P_2,4,4,4_][DEP] + thiophene + heptane					
0.002	0.000	0.544	0.000	–	–
0.002	0.016	0.515	0.043	2.69	6.0
0.004	0.043	0.443	0.104	2.42	5.1
0.005	0.079	0.376	0.170	2.15	4.3
0.008	0.112	0.323	0.214	1.91	3.6
0.009	0.148	0.274	0.257	1.74	3.1
0.008	0.172	0.244	0.280	1.63	2.8
0.010	0.199	0.201	0.301	1.51	2.4
0.013	0.212	0.187	0.309	1.46	2.2
0.013	0.239	0.160	0.326	1.36	2.0
[P_2,4,4,4_][DEP] + benzothiophene + heptane					
0.002	0.000	0.548	0.000	–	–
0.006	0.015	0.495	0.062	4.13	9.1
0.007	0.044	0.418	0.163	3.70	8.4
0.009	0.068	0.369	0.224	3.29	7.5
0.006	0.092	0.327	0.277	3.01	6.9
0.011	0.120	0.287	0.323	2.69	6.0
0.006	0.167	0.218	0.380	2.28	4.7
0.014	0.241	0.171	0.422	1.75	3.2
0.014	0.271	0.147	0.435	1.61	2.7
0.015	0.311	0.115	0.453	1.46	2.3

Standard uncertainties are: *u*(*x*) < 0.003, *u*(*T*) = 0.05 K

The solubility of thiophene at *T* = 308.15 K is equal to $x_{2}^{\text{IL}}$ **=** 0.814 for [C_1_C_5_PIP][NTf_2_] (*x*_2_^IL^ **=** 0.797 for [C_1_C_3_PIP][NTf_2_] at *T* = 298.15 K [18], the influence of temperature is minimal; the largest solubility of thiophene in the piperidinium-based IL was observed for C_1_C_6_PIP][NTf_2_] [18]). Complete miscibility with thiophene was observed for [P_2,4,4,4_][DEP].

In the binary system with benzothiophene, the solubility of benzothiophene in [C_1_C_5_PIP][NTf_2_] at *T* = 308.15 K is equal to $x_{2}^{\text{IL}}$ **=** 0.873 ($x_{2}^{\text{IL}}$ **=** 0.945 for [C_1_C_3_PIP][NTf_2_] at *T* = 308.15 K [18]. Complete miscibility with benzothiophene was observed for [P_2,4,4,4_][DEP].

Immiscibility is observed in the {thiophene, or benzothiophene (2) + heptane (3)} binary mixture, as was reported previously [18].

**Fig. 1:**
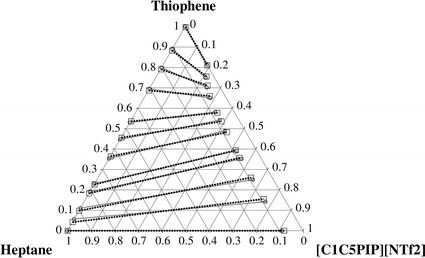
Plot of the experimental (*filled circle*, *gray solid lines*) results versus values calculated with the NRTL equation (*square*, *black dotted lines*) for the composition tie lines of the ternary system {[C_1_C_5_PIP][NTf_2_] (1) + thiophene (2) + heptane (3)} at *T* = 308.15 K

**Fig. 2:**
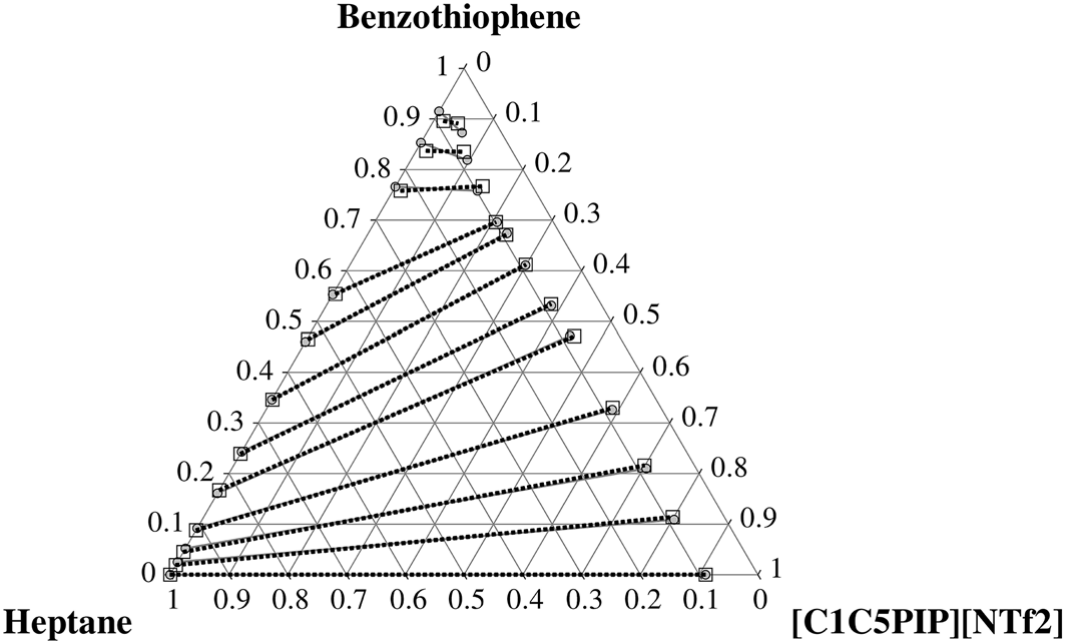
Plot of the experimental (*filled circle*, *gray solid lines*) results versus values calculated with the NRTL equation (*square*, *black dotted lines*) for the composition tie lines of the ternary system {[C_1_C_5_PIP][NTf_2_] (1) + benzothiophene (2) + heptane (3)} at *T* = 308.15 K

**Fig. 3:**
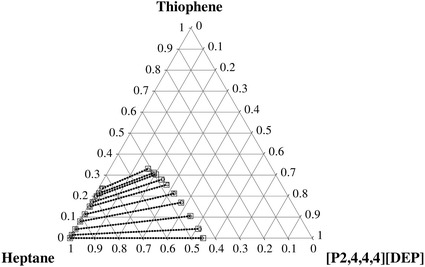
Plot of the experimental (*filled circle*, *gray solid lines*) results versus values calculated with the NRTL equation (*square*, *black dotted lines*) for the composition tie lines of the ternary system {[P_2,4,4,4_][DEP] (1) + thiophene (2) + heptane (3)} at *T* = 308.15 K

**Fig. 4:**
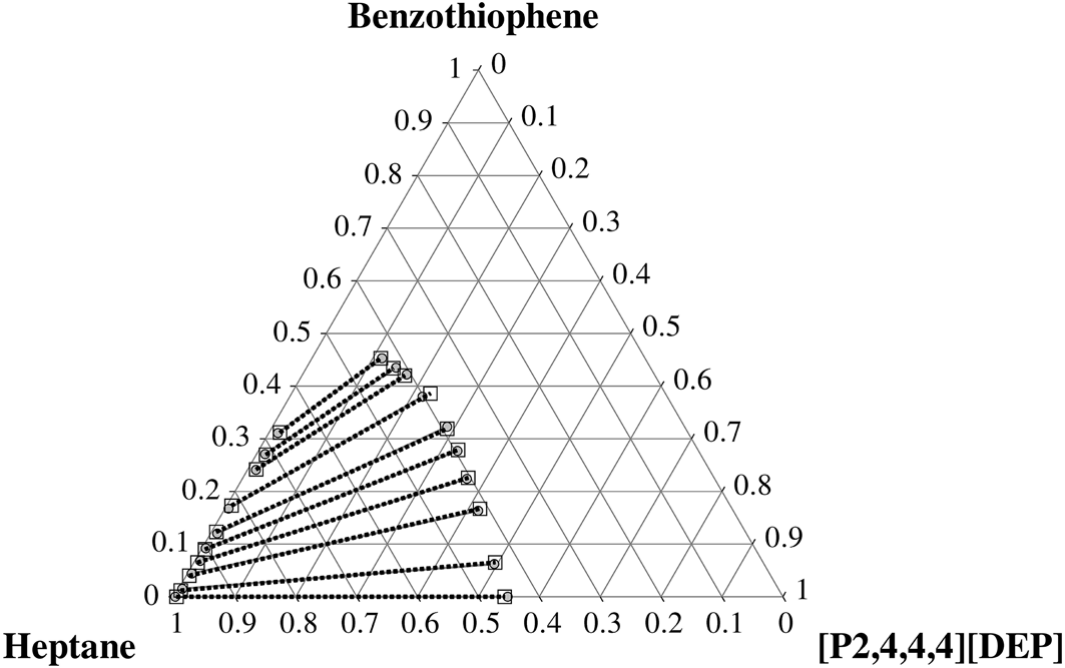
Plot of the experimental (*filled circle*, *gray solid lines*) results versus values calculated with the NRTL equation (*square*, *black dotted lines*) for the composition tie lines of the ternary system {[P_2,4,4,4_][DEP] (1) + benzothiophene (2) + heptane (3)} at *T* = 308.15 K

The determined experimental tie-lines for the ternary LLE systems are plotted in Figs. 1, 2, 3, 4 for thiophene and benzothiophene, respectively. Figures 1, 2, 3, 4 show that the two-phase region is much larger for [C_1_C_5_PIP][NTf_2_] than that for [P_2,4,4,4_][DEP].

**Fig. 5:**
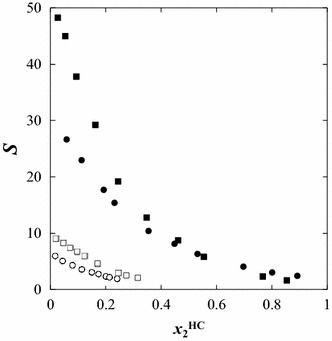
Plot of the selectivity (*S*) as a function of the mole fraction of solute in the hydrocarbon-rich phase for the ternary systems: *closed circle* {[C_1_C_5_PIP][NTf_2_] (1) + thiophene (2) + heptane (3)}, *filled square* {[C_1_C_5_PIP][NTf_2_] (1) + benzothiophene (2) + heptane (3)}, *open circle* {[P_2,4,4,4_][DEP] (1) + thiophene (2) + heptane (3)}, and *open square* {[P_2,4,4,4_][DEP] (1) + benzothiophene (2) + heptane (3)}, at *T* = 308.15 K

**Fig. 6:**
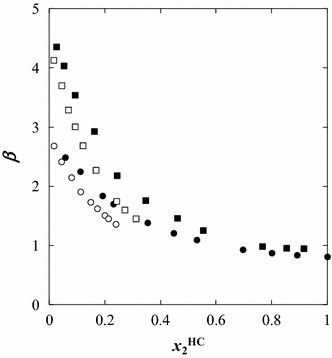
Plot of the solute distribution ratio (*β*) as a function of the mole fraction of solute in the hydrocarbon-rich phase for the ternary systems: *closed circle* {[C_1_C_5_PIP][NTf_2_] (1) + thiophene (2) + heptane (3)} *filled square* {[C_1_C_5_PIP][NTf_2_] (1) + benzothiophene (2) + heptane (3)} *open circle* {[P_2,4,4,4_][DEP] (1) + thiophene (2) + heptane (3)}, and *open square* {[P_2,4,4,4_][DEP] (1) + benzothiophene (2) + heptane (3)}, at *T* = 308.15 K

The results obtained in this work show that the more suitable IL for the separation of thiophene, or benzothiophene from heptane, is [C_1_C_5_PIP][NTf_2_] because of its much larger selectivity (*S*) and the comparable solute distribution ratio (*β*). These parameters are defined as follows:

1 $$ \beta = \frac{{x_{ 2}^{\text{II}} }}{{x_{ 2}^{\text{I}} }} $$

2 $$ S = \frac{{x_{ 2}^{\text{II}} \cdot x_{ 3}^{\text{I}} }}{{x_{ 2}^{\text{I}} \cdot x_{ 3}^{\text{II}} }} $$

where *x* is the mole fraction; superscripts I and II refer to the heptane-rich phase and the IL-rich phase, respectively. Subscripts 2 and 3 refer to the sulfur compound and heptane, respectively. The values of *β* and *S* are listed in Table 2 for thiophene and benzothiophene. Figures 5 and 6 present measured values of *β* and *S* for ILs for thiophene and benzothiophene.

The values presented in Table 2 show that the distribution ratio coefficient are in the range of 0.81–2.41, 0.95–4.36, 1.36–2.69 and 1.46–4.13 for [C_1_C_5_PIP][NTf_2_]/thiophene, [C_1_C_5_PIP][NTf_2_]/benzothiophene, [P_2,4,4,4_][DEP]/thiophene and [P_2,4,4,4_][DEP]/benzothiophene, respectively.

The selectivities of the separation in the system thiophene or benzothiophene/heptane is quite high for [C_1_C_5_PIP][NTf_2_] and very low for [P_2,4,4,4_][DEP]. The values listed in Table 2 for the best tie-lines are: 26.7, 48.3, 6.0 and 9.1 for [C_1_C_5_PIP][NTf_2_]/thiophene, [C_1_C_5_PIP][NTf_2_]/benzothiophene, [P_2,4,4,4_][DEP]/thiophene and [P_2,4,4,4_][DEP]/benzothiophene, respectively.

In this work the effect of the alkane chain length on the cation of the piperidinium-based IL was examined for comparison with previously measured data for [C_1_C_3_PIP][NTf_2_]/thiophene, [C_1_C_4_PIP][NTf_2_]/thiophene, and [C_1_C_6_PIP][NTf_2_]/thiophene at *T* = 298.15 K, and of [C_1_C_3_PIP][NTf_2_]/benzothiophene at *T* = 308.15 K [18] (the influence of temperature is not large). The effect of anion in the phosphonium-based IL was also verified. The characteristic extraction parameters obtained in this work are compared to the few previously described in the open literature in Table 3. Unfortunately, the selectivity for [C_1_C_5_PIP][NTf_2_] obtained in this work is slightly worse than that for [C_1_C_3_PIP][NTf_2_] measured by us earlier [18]. The values of selectivity presented for 1-alkylcyanopyridinium-based ILs at *T* = 308.15 K measured in our earlier work [16] are also larger than those for piperidinium-based ILs [18] (see Table 3).

**Table 3:** Comparison of solute distribution ratio (*β*) and selectivity (*S*) for sulfur compounds extraction

IL	LLE system	*T*/K	*β*_max_	*S*_max_	Ref.
[C_1_C_5_PIP][NTf_2_]	IL + thiophene + heptane	308	2.49	26.7	This work
[C_1_C_5_PIP][NTf_2_]	IL + benzothiophene + heptane	308	4.36	48.3	This work
[C_1_C_3_PIP][NTf_2_]	IL + thiophene + heptane	298	2.50	60.3	[18]
[C_1_C_3_PIP][NTf_2_]	IL + benzothiophene + heptane	308	5.36	93.0	[18]
[COC_2_MPIP][NTf_2_]^a^	IL + thiophene + heptane	298	2.64	62.9	[14]
[COC_2_MPIP][FAP]^b^	IL + thiophene + heptane	298	4.00	56.8	[13]
[BCN^4^Py][NTf_2_]^c^	IL + thiophene + heptane	308	1.93	62.2	[16]
[BCN^4^Py][NTf_2_]^c^	IL + benzothiophene + heptane	308	3.50	117.1	[16]
[P_2,4,4,4_][DEP]	IL + thiophene + heptane	308	2.69	6.0	This work
[P_2,4,4,4_][DEP]	IL + benzothiophene + heptane	308	4.13	9.1	This work
[EMIM][DEP]^d^	IL + thiophene + hexane	298	2.88	48.8	[12]
[P_1,4,4,4_][CH_3_SO_4_]	IL + thiophene + cyclohexane	298	1.38	5.38	[20]
[DMIM][MP]^e^	IL + thiophene + heptane	298	0.42	1,756	[7]

^a^1-(2-Methoxyethyl)-1-methylpiperidinium bis{(trifluoromethyl)sulfonyl}imide

^b^1-(2-Methoxyethyl)-1-methylpiperidinium trifluorotris(perfluoroethyl)phosphate

^c^1-Butyl-4-cyanopyridinium bis{(trifluoromethyl)sulfonyl}imide

^d^1-Ethyl-3-methylimidazolium diethylphosphate

^e^1,3-Dimethylimidazolium methylphosphonate

The selectivities for [C_1_C_3_PIP][NTf_2_] are comparable to those for 4-(2-methoxyethyl)-4-methylpiperidinium trifluorotris(perfluoroethyl)phosphate [COC_2_MPIP][FAP] ILs at *T* = 298.15 K [13], or to 4-(2-methoxyethyl)-4-methylpiperidinium bis{(trifluoromethyl)sulfonyl}imide [COC_2_MPIP][NTf_2_] [14]. For further comparisons see our earlier work [16].

The extraction results for [P_2,4,4,4_][DEP] are very low and similar to [P_1,4,4,4_][CH_3_SO_4_] [20]. It can be definitely concluded that phosphonium-based cations are not suitable for these separation processes. However, for the diethylphosphate anion [DEP]^−^ and imidazolium-based cation [EMIM]^+^, the results are comparable to those obtained in this work with [C_1_C_5_PIP][NTf_2_] but with a lower *β* value [18] (see Table 3).

It can be also seen from Figs. 5 and 6 that *β* and *S* decrease as the solute mole fraction (thiophene, or benzothiophene) in the heptane phase increases, for all systems, when going through the tie-line end compositions.

## Data Correlation

The ternary LLE data measured in this study were correlated (the tie-line correlation) using the well known non-random liquid equation, NRTL [23]. The equations and algorithms used for the calculation of the compositions in both phases follow the method described by Walas [24]. The objective function *F*(*P*) was used to minimize the difference between the experimental and calculated compositions:

3 $$ F(P) = \sum\limits_{i = 1}^{n} {\left[ {x_{2}^{{{\text{I,}}exp}} - x_{ 2}^{\text{I,calc}} \left( {PT} \right)} \right]^{2} + \left[ {x_{3}^{{{\text{I,}}exp}} - x_{3}^{\text{I,calc}} \left( {PT} \right)} \right]}^{2} + \left[ {x_{2}^{{{\text{II,}}exp}} - x_{ 2}^{\text{II,calc}} \left( {PT} \right)} \right]^{2} + \left[ {x_{3}^{{{\text{II,}}exp}} - x_{ 3}^{\text{II,calc}} \left( {PT} \right)} \right]^{2} $$

where *P* is the set of parameters vector, *n* is the number of experimental points, $x_{2}^{{{\text{I}},exp}}$, $x_{3}^{{{\text{I}},exp}}$ and $x_{ 2i}^{{{\text{I}},{\text{calc}}}} \left( {PT} \right)$, $x_{3}^{\text{I,calc}} \left( {PT} \right)$ are the experimental and calculated mole fractions of one phase, and $x_{2}^{{{\text{II}},exp}}$, $x_{3}^{{{\text{II,}}exp}}$ $x_{ 2}^{\text{II,calc}} \left( {PT} \right)$, and $x_{ 3}^{\text{II,calc}} \left( {PT} \right)$ are the experimental and calculated mole fractions of the second phase. The binary parameters of each constituent were regressed by minimizing the sum of the squares of the differences between the experimental and calculated mole fractions of each component of both liquid phases for each ternary system. These binary parameters were obtained for all data simultaneously (binaries and ternaries).

The value of the non-randomness parameter, *α*_*ij*_, was optimized in order to obtain the best model fit. The correlated parameters are given in Table 4 along with the root mean square deviations (RMSD). The RMSD values, which are a measure of the precision of the correlation, were calculated according the equation:

4 $$ {\text{RMSD}} = \left( {\sum\limits_{i} {\sum\limits_{l} {\sum\limits_{m} {\left[ {x_{ilm}^{exp} - x_{ilm}^{\text{calc}} } \right]^{2} /6k} } } } \right)^{1/2} $$

where *x* is the mole fraction and the subscripts *i, l*, and *m* designate the component, phase, and tie-line, respectively. The Rosenbrock simplex method was used in an effort to minimize the objective function. The compositions calculated from the correlations are included in Figs. 1 to 4. The correlation results, obtained for the four systems studied, are satisfactory. The experimental and calculated LLE data agreed relatively well.

**Table 4:** Binary interaction parameters, parameter *α*_*ij*_ and root mean square deviation (*σ*_*x*_) for the NRTL equation for the ternary systems {[C_1_C_5_PIP][NTf_2_] or [P_2,4,4,4_][DEP] (1) + thiophene or benzothiophene (2) + heptane (3)} at *T* = 308.15 K, *p* = 101.33 kPa

*ij*	Δ*g*_12_/(J·mol^−1^)	Δ*g*_21_/(J·mol^−1^)	*α*_*ij*_	RMSD *σ*_*x*_
[C_1_C_5_PIP][NTf_2_] + thiophene + heptane				
12	–8261.11	23225.94	0.2	0.007
13	2536.43	15361.68		
23	–460.97	475.45		
[C_1_C_5_PIP][NTf_2_] + benzothiophene + heptane				
12	–5698.73	12608.56	0.2	0.007
13	5142.95	34672.57		
23	2498.84	2232.66		
[P_2,4,4,4_][DEP] + thiophene + heptane				
12	–5566.06	7459.65	0.2	0.003
13	–2785.55	19670.14		
23	–3351.22	6008.81		
[P_2,4,4,4_][DEP] + benzothiophene + heptane				
12	–9265.59	11427.53	0.2	0.004
13	–2823.06	16531.68		
23	–1048.01	2808.72		

## Conclusions

The ternary liquid–liquid phase equilibrium data were measured in this study for the extraction of thiophene or benzothiophene from heptane using two ILs. Four ternary systems {IL + thiophene or benzothiophene + heptane} were analytically determined using GC for the composition analysis at temperature *T* = 308.15 K at ambient pressure. It has been demonstrated that the 1-pentyl-1-methylpiperidinium bis{(trifluoromethyl)sulfonyl}imide IL is much more effective than the phosphonium-based IL for extraction of thiophene or benzothiophene from alkanes. Sulfur compounds can be extracted easily by piperidinium-based ILs, leading to low sulfur content in fuels. Our earlier experimental results revealed that the solubility of sulfur compounds in the IL increases as the alkyl chain length increases [18]. The capacity of extraction, described in terms of the selectivity and the solute distribution ratio coefficients, was calculated for all ternary systems and compared to the published data used in similar extraction problems. Based on the values obtained, [C_1_C_5_PIP][NTf_2_] was found to be useful for the extraction of sulfur compounds from alkanes; however, it is not as good as [C_1_C_3_PIP][NTf_2_] measured previously [18]. The selectivity and the solute distribution ratio decrease as the mole fraction of thiophene or benzothiophene in the heptane-rich phase increases. The best selectivity (*S*) is observed for very low mole fractions of S-compounds in the hydrocarbon-rich phase $(x_{2}^{\text{HC}} \, = \,0.0 5)$ (see Fig. 5), which may be compared with the results of the HDS method for the removal of the S-compounds. The experimental data in this work was regressed using the NRTL activity coefficient model and binary interaction parameters. The non-randomness parameter was also determined through the reduction of the experimental data. The model exhibited an excellent fit to the data with the average RMSD values between 0.003 and 0.007.

## Electronic Supplementary Material

Below is the link to the electronic supplementary material.

Supplementary material 1 (DOC 50 kb)
