# Supplementary material for: Ternary Liquid–Liquid Equilibria for Mixtures of {Ionic Liquid + Thiophene or Benzothiophene + Heptane} at T = 308.15 K
Source: J Solution Chem. 2014 Dec 23;44(3):382–94. doi: 10.1007/s10953-014-0276-y (PMC4412552; doi:10.1007/s10953-014-0276-y)
Supplement: Supplementary file 1 — Supplementary material 1 (DOC 50 kb) [file 10953_2014_276_MOESM1_ESM.doc]

**Supporting Information for**

**Ternary Liquid-Liquid Equilibria for Mixtures of {Ionic Liquid + Thiophene or Benzothiophene + Heptane} at *T* = 308.15 K**

**Urszula Domańska · Klaudia Walczak**

Department of Physical Chemistry, Faculty of Chemistry, Warsaw University of Technology, Noakowskiego 3, 00-664 Warsaw, Poland

**Table 1S** The sources, mass fraction purities, purification method, water content, measured and literature densities of materials used

| Chemical name/  CAS No | Source | Initial mass fraction purity | Purification method | Analysis method | Final mass fraction purity | Water  content/ mass fraction | Expt. density  *ρ*/g⋅cm^–3^  (298.15; 101.33 kPa) | Lit. density  *ρ*/g⋅cm^–3^  (298.15; 101.33 kPa) |
| --- | --- | --- | --- | --- | --- | --- | --- | --- |
| 1-Pentyl-1-methylpiperidinium bis{(trifluoromethyl)sulfonyl}imide | Iolitec | ≥ 0.990 | Low pressure 24 h  300 K | Water content,  density | - | 250 × 10^–6^ | 1.35016 | 1.35013^a^ |
| Tributylethylphosphonium diethylphosphate  CAS: 20445-94-7 | Iolitec | ≥ 0.95 | Low pressure 24 h  300 K | Water content,  density | - | 250 × 10^–6^ | 1.0089 | 1.00876^b^ |
| Thiophene  CAS No. 110-02-1 | Merck | ≥ 0.990 | distillation | GLC  Water content  density | 0.9988 (GC) | 290 × 10^–6^ | 1.05892 | 1.05887^c^ |
| Bezothiophene  CAS No. 95-15-8 | Merck | ≥ 0.995 | distillation | GLC  Water content, density | 0.9950 (GC) | 290 × 10^–6^ | 1.14270 | 1.15055 (308.15)^d^ |
| Heptane  CAS No. 142-82-5 | Merck | ≥ 0.990 | distillation | GLC  Water content, density | 0.9979 (GC) | 260 × 10^–6^ | 0.67954 | 0.67946^e^ |
| Propan-1-ol  CAS No. 71-23-8 | Aldrich | ≥ 0.990 | – | Water content,  density | 0.9988 (GC) | 190 × 10^–6^ | 0.79952 | 0.79960^e^ |
| Acetone  CAS No : 67-64-1 | Aldrich | ≥ 0.995 | – | Water content, density | 0.9997 (GC) | 220 × 10^–6^ | 0.78532 | 0.78508^c^ |

*u*(*ρ*) = ±0.00005 g∙cm^–3^, *u*(*T*) = ±0.02 K, u(*p*) = ±0.1 kPa.

^a^ Paduszyński, K., Domańska, U.: Experimental and theoretical study on infinite dilution activity coefficients of various solutes in piperidinium ionic liquids. J. Chem. Thermodyn. **60**, 169–178 (2013)

^b^ Deive, F.J., Rivas, M.A., Rodríguez, A.: Study of thermodynamic and transport properties of phosphonium-based ionic liquids. J. Chem. Thermodyn. **62**, 98–103 (2013)

^c^ KNOVEL DIPPR 801

^d^ Domańska, U., Walczak, K., Zawadzki, M.: Separation of sulfur compounds from alkanes with 1-alkylcyanopyridinium-based ionic liquids. J. Chem Thermodyn. **69**, 27–35 (2014)

^e^ Riddick, A., Bunger, W. B., Sakano, T. K.: Organic Solvents, Physical Properties and Method of Purification, 4th edn. Wiley-Interscience: New York (1986)

**Table 2S** Operational conditions in the gas chromatograph for compositional analysis of the phases in equilibrium

| Element | Characteristic | Description |
| --- | --- | --- |
| Columns | Type | Elit-5 PerkinElmer DB-5 (5% diphenyl/95%  dimethyl polysiloxane), length 30 m, inner diameter 0.53 mm, film thickness 1.5 μm  Elite-Wax PerkinElmer, length 30 m, inner diameter 0.53 mm, film thickness, 1.0 μm |
|  | Flow | 5 mL∙min^–1^ |
|  | Carrier gas | Helium |
| Oven | Temperature | 343.15 K |
| Injector | Injection volume | 0.1 μL |
|  | Split ratio | 10:1 |
|  | Temperature | 423 K |
| Detector | Type | Flame ionization detector (FID) |
|  | Temperature | 493 K |
